# Thrombolysis in Prosthetic Valve Thrombosis: A Safe and Effective Alternative?

**DOI:** 10.7759/cureus.94043

**Published:** 2025-10-07

**Authors:** Mohamed Samy Lebbar, Fatimazahra Merzouk, Mohamed Ghali Benouna

**Affiliations:** 1 Cardiology, Cheikh Khalifa International University Hospital, Mohammed VI University of Health Sciences, Casablanca, MAR; 2 Cardiology, Mohammed VI International University Hospital, Mohammed VI University of Health Sciences, Casablanca, MAR; 3 Cardiology, Cheikh Khalifa International University Hospital, Mohammed VI University of Health and Sciences, Casablanca, MAR

**Keywords:** anticoagulation, echocardiography, mechanical heart valve, prosthetic valve thrombosis, thrombolysis

## Abstract

Prosthetic valve thrombosis (PVT) is a rare but life-threatening complication of mechanical heart valves. Surgery remains the standard of care, but thrombolysis has emerged as a valuable alternative in patients with high surgical risk or refusal. We describe a 38-year-old woman, five years after double mitral-aortic mechanical valve replacement, who had discontinued anticoagulation one month prior to admission. She presented with acute angina-like chest pain and severe dyspnea (New York Heart Association (NYHA) class IV). Clinical examination revealed diminished prosthetic clicks and pulmonary rales, while echocardiography and fluoroscopy confirmed obstruction of one aortic prosthetic leaflet with high transvalvular gradients. Coronary angiography showed a distal left anterior descending (LAD) artery occlusion, most likely embolic, with otherwise normal vessels.

The diagnosis of obstructive aortic prosthetic thrombosis complicated by pulmonary edema and embolic ST-segment elevation myocardial infarction (STEMI) was made. As the patient declined surgical intervention, intravenous thrombolysis with tenecteplase (100 mg protocol plus unfractionated heparin) was initiated, along with diuretics. Within one hour, prosthetic sounds normalized, and echocardiography showed marked gradient reduction, with no complications. At the three-month follow-up, echocardiography remained satisfactory.

This case highlights thrombolysis as a safe and effective therapeutic option in selected patients with obstructive PVT, particularly those unsuitable for or refusing surgery, provided that diagnosis is imaging-based and management involves close monitoring and multidisciplinary decision-making.

## Introduction

Prosthetic valve thrombosis (PVT) is a severe complication of mechanical valves with potentially fatal consequences. This condition occurs when a blood clot obstructs the mechanical valve, impairing leaflet motion and increasing the transvalvular pressure gradient (the pressure difference across the valve). Patients often present with heart failure symptoms, graded by the New York Heart Association (NYHA) classification, where class IV indicates severe shortness of breath at rest. The therapeutic goal is to promptly restore valve function and prevent embolic complications.

Despite anticoagulation therapy, some patients develop obstructive thrombosis requiring urgent management; redo surgery carries substantial perioperative risk (≈ 8%-30%) depending on urgency and comorbidities [1]. Over the last decade, comparative evidence between surgery and thrombolysis, spanning meta-analyses, cohorts, and systematic reviews, has expanded significantly, showing at least comparable efficacy for thrombolysis and a signal toward lower early mortality in selected patients [2-4].

Guideline positions remain divergent, with the American College of Cardiology/American Heart Association (ACC/AHA) acknowledging thrombolysis as an initial option in selected cases, whereas European Society of Cardiology/European Association for Cardio-Thoracic Surgery (ESC/EACTS) recommendations still favor surgery for left-sided obstructive PVT, particularly in the aortic position [5, 6]. These data provide the rationale for considering thrombolysis in carefully selected patients and frame contemporary practice patterns, particularly as optimized “ultra-slow” infusion regimens, prolonged, low-dose thrombolytic infusions, have substantially improved safety.

## Case presentation

History

A 38-year-old woman (BMI 20 kg/m²), mother of three, with a history of double mechanical mitral-aortic valve replacement for rheumatic disease five years earlier, presented with acute chest pain for 12 hours and dyspnea at rest (NYHA class IV). She admitted to discontinuing vitamin K antagonists one month earlier due to personal difficulties.

Clinical examination

The patient's vitals were as follows: blood pressure 132/76 mmHg, heart rate 96 beats per minute (bpm), and oxygen saturation 99%. A cardiovascular exam revealed diminished prosthetic clicks, a systolic murmur, and pulmonary rales. The neurological exam was normal.

Investigations

**Figure 1:**
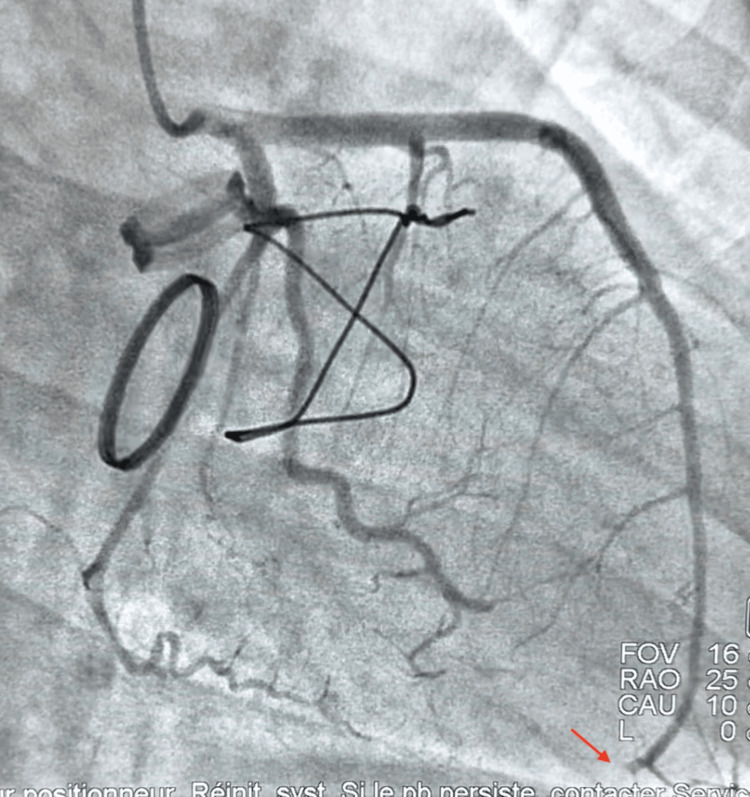
Coronary angiographic view revealing abrupt cutoff of the distal LAD compatible with embolic occlusion (red arrow). This finding supports the diagnosis of embolic coronary obstruction secondary to prosthetic valve thrombosis. LAD: left anterior descending

The electrocardiogram (ECG) showed ST-segment elevation in the antero-septal-lateral leads. Coronary angiography revealed an acute occlusion of the distal left anterior descending (LAD) artery, which was highly suggestive of an embolic origin (Figure 1).

**Figure 2:**
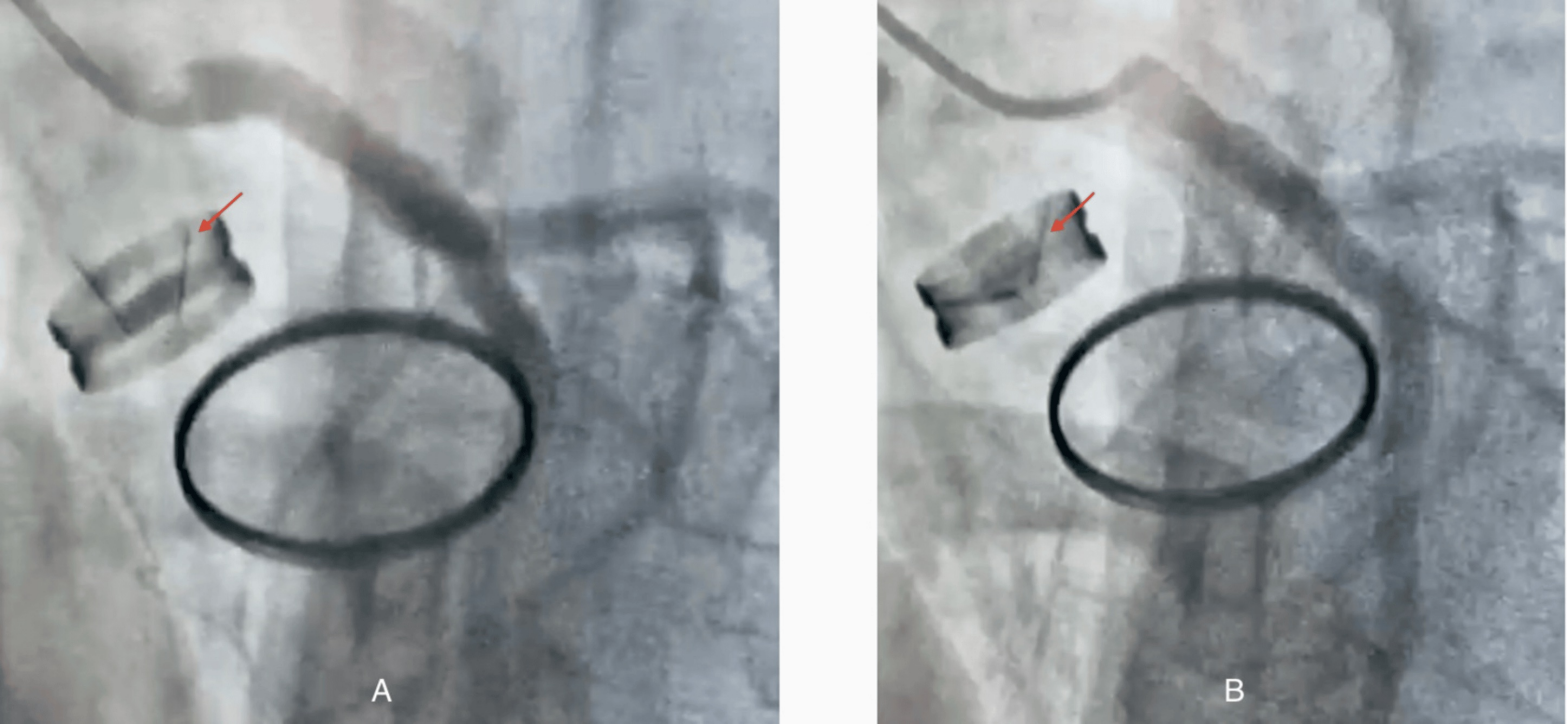
Fluoroscopy demonstrating immobility of one leaflet of the mechanical aortic prosthesis (red arrow); (A) systole, (B) diastole. The absence of leaflet excursion is consistent with a thrombus obstructing prosthetic motion.

Fluoroscopy demonstrated immobility of one of the aortic prosthetic leaflets (Figure 2).

**Figure 3:**
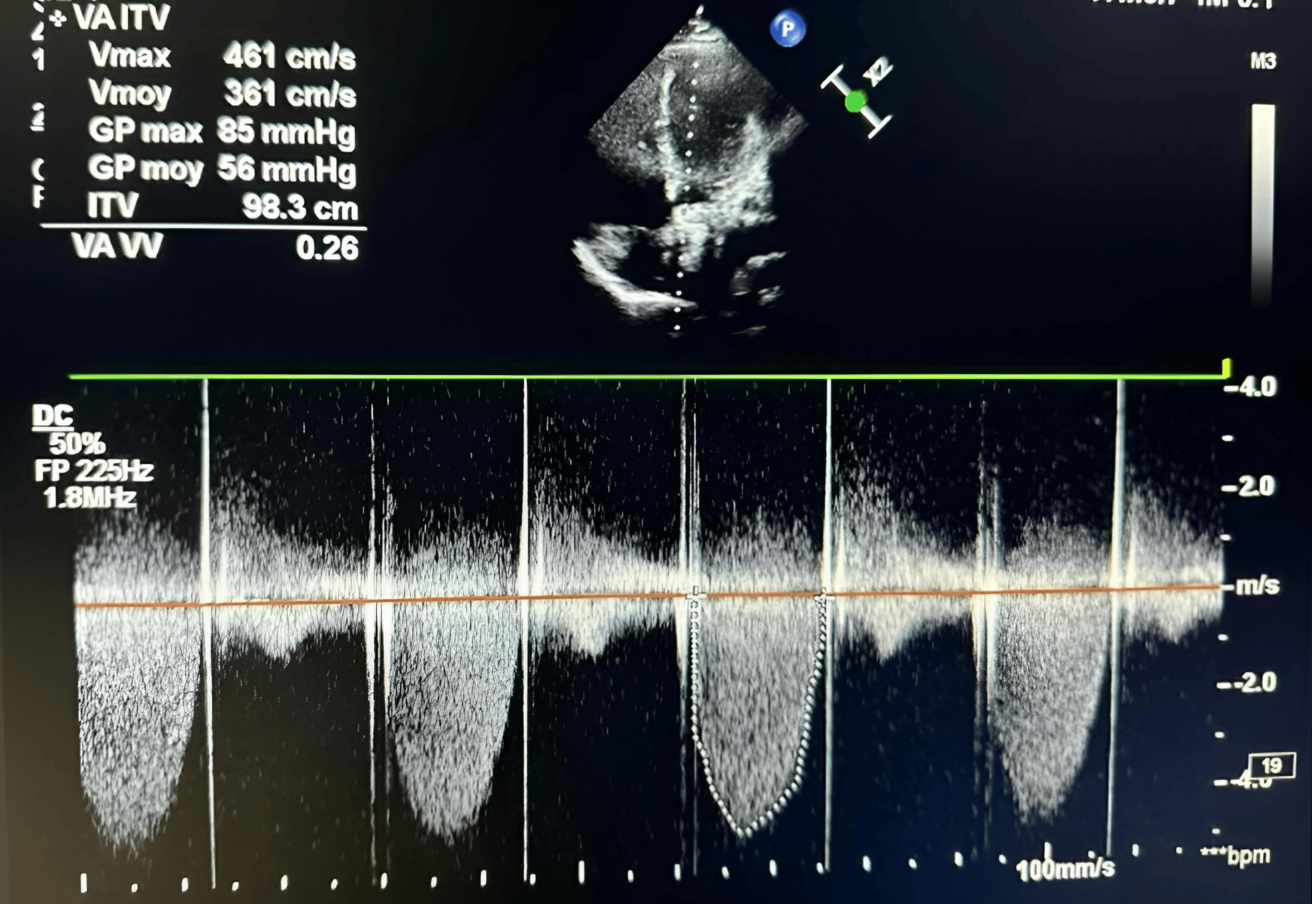
Continuous-wave Doppler illustrating high-velocity flow through the aortic prosthesis, suggesting leaflet obstruction. The markedly elevated gradient (56 mmHg) indicates severe obstruction consistent with prosthetic valve thrombosis.

Echocardiography revealed a mean transvalvular gradient of 56 mmHg and a valve area of 0.65 cm², along with elevated left ventricular (LV) filling pressures and preserved systolic function. The imaging window was suboptimal, which limited direct visualization of the thrombus (Figure 3).

Laboratory results were as follows: international normalized ratio (INR) 1.12 (range: 2.5-3.5), troponin 1.12 ng/mL, D-dimers 2513 ng/mL, CRP 50 mg/L, hemoglobin (Hb) 11 g/dL (Table 1).

**Table 1:** Laboratory test results

Parameter	Patient's values	Reference range	Units
International normalized ratio (INR)	1.12	2.5–3.5 (mechanical valve)	–
Troponin	1.12	< 0.04	ng/mL
D-dimers	2513	< 500	ng/mL
C-reactive protein (CRP)	50	< 5	mg/L
Hemoglobin	11	12–16 (female)	g/dL

Diagnosis

This case was diagnosed as obstructive thrombosis of the aortic prosthesis complicated by pulmonary edema and acute distal LAD embolic occlusion presenting as ST-segment elevation myocardial infarction (STEMI).

Management

As per the 2021 ESC/EACTS guidelines, urgent surgery was recommended, but the patient declined. Thrombolysis was performed using tenecteplase (10 mg bolus + 90 mg infusion over 90 minutes) with unfractionated heparin and diuretics.

Outcome

Within one hour, prosthetic sounds normalized; echocardiography showed gradient reduction (56 → 23 mmHg), and leaflet mobility was restored on fluoroscopy. No complications occurred. Following successful thrombolysis, the patient was discharged on long-term oral anticoagulation with a vitamin K antagonist (warfarin), with reinforced INR monitoring to maintain therapeutic range.

**Figure 4:**
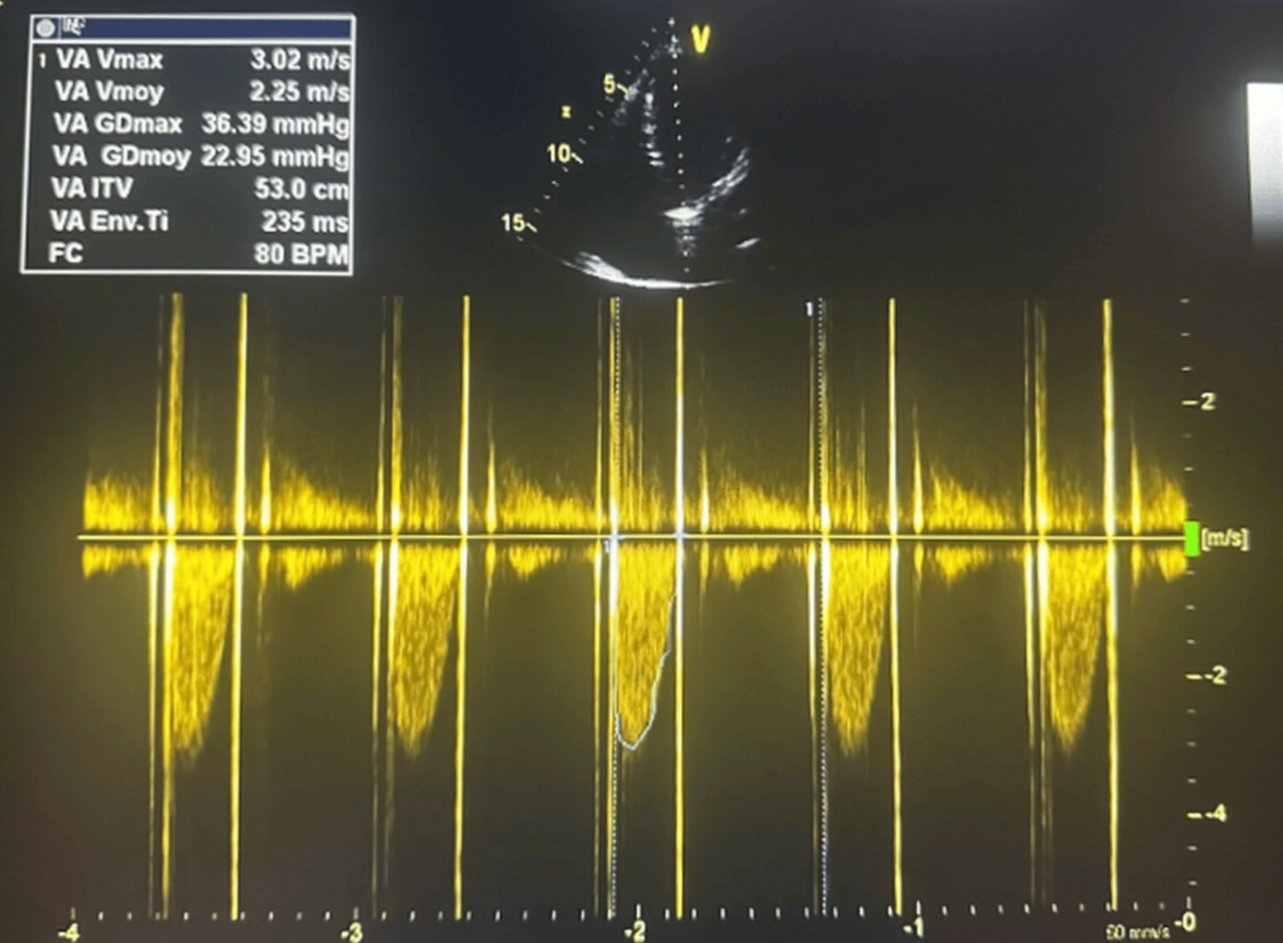
Doppler echocardiography after thrombolysis demonstrating normalization of transaortic gradients; Restoration of normal flow confirms successful thrombolytic resolution of the obstruction.

**Figure 5:**
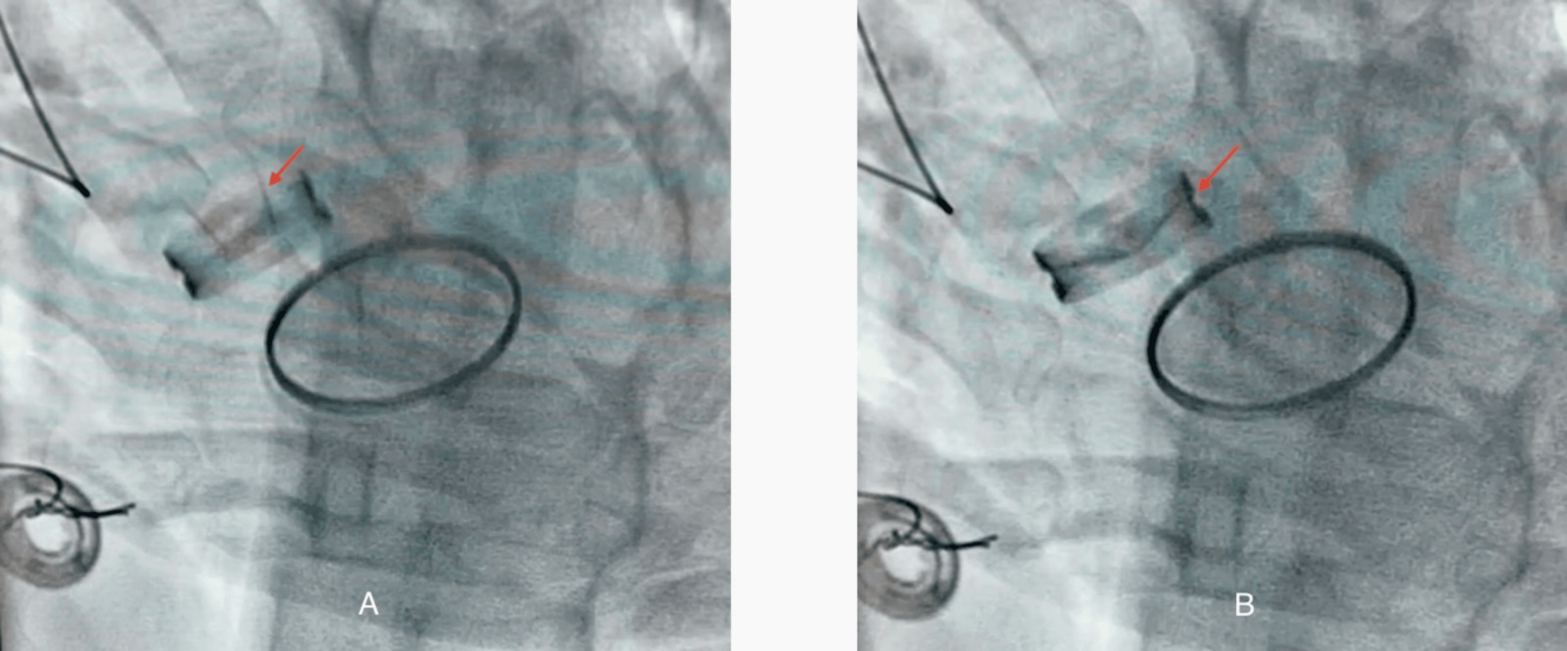
Fluoroscopy after thrombolysis showing restored mobility of the previously blocked aortic prosthetic leaflet (red arrow); (A) systole, (B) diastole. The reappearance of normal leaflet motion confirms full mechanical recovery after thrombolytic therapy.

At three months, echocardiography confirmed durable improvement and full prosthetic mobility (Figures 4, 5).

Key diagnostic clues

The key diagnostic clues were as follows: recent discontinuation of anticoagulation (one month off vitamin K antagonist); acute chest pain (12 hours) + NYHA IV dyspnea; diminished prosthetic clicks and pulmonary rales; high transvalvular gradient (56 mmHg) on echocardiography; immobile aortic leaflet on fluoroscopy; distal LAD embolic occlusion on angiography.

## Discussion

Surgical reoperation has long been regarded as the gold standard for obstructive PVT, but operative mortality remains substantial in urgent settings, historically ≈ 8%-30%, depending on timing, valve position, and comorbidities [1]. This high upfront risk has fueled interest in fibrinolysis as a less invasive alternative. Early observational work, such as Roudaut et al., established feasibility while underscoring the need to minimize embolic and hemorrhagic complications [7]. Over time, refinements in selection and dosing reshaped the risk-benefit profile, and comprehensive reviews have positioned thrombolysis as a legitimate option within structured protocols [4].

Comparative syntheses consistently support this shift. Meta-analyses and systematic reviews demonstrate that thrombolysis achieves clinical success rates similar to surgery while offering reduced early mortality in appropriately selected populations [2-4]. In aggregate series, thrombolysis yields ≈ 80%-90% success with mortality ≈ 6%-8%, versus 15%-20% for urgent surgery, figures that, though heterogeneous, are consistent across analyses [2-4]. Complications such as embolism and bleeding have declined with protocol optimization.

The pivotal advance has been low-dose, slow/ultra-slow infusion regimens of tissue-type plasminogen activator. Comparison of Different TEE-Guided thrombolytic Regimens for prosthetic valve thrombosis (TROIA) trial showed > 90% success with < 6% complication rate [8]. PROsthetic MEchanical valve Thrombosis and the prEdictors of outcomE (PROMETEE) trial confirmed that slower infusions mitigate bleeding and embolic risk [9]. Other studies [10, 11] confirm reproducibly high success and safety.

In the present case, a standard-dose tenecteplase protocol was used rather than an ultra-slow infusion regimen. This choice reflected the need for rapid reperfusion given concomitant embolic coronary occlusion and severe hemodynamic compromise. Although low-dose, prolonged infusions are associated with lower bleeding risk, the accelerated regimen achieved prompt valve recovery without complications, underscoring the importance of tailoring the thrombolytic strategy to clinical urgency and stability.

Real-world evidence (e.g., Khan et al. [12]) supports reproducible results outside specialized centers, including resource-limited settings.

Guideline recommendations remain heterogeneous. The ACC/AHA recognizes thrombolysis as acceptable initial therapy in selected obstructive PVT cases [5], whereas the ESC/EACTS favors surgery, particularly for aortic prostheses [6]. Given evolving data, individualized decision-making within a multidisciplinary heart team is essential.

Another consideration is coronary artery obstruction from thromboembolism, a rare but severe complication of PVT. Its occurrence may strongly influence treatment choice: patients with acute coronary occlusion and ongoing ischemia may require surgical intervention, whereas in the absence of such embolic events, thrombolysis remains a valid, less invasive option. This highlights the importance of rapid imaging and careful assessment of embolic complications when tailoring management.

Special clinical contexts (e.g., pregnancy) have shown the feasibility of low-dose, slow infusions [13]. Across all settings, two determinants emerge for durable benefit: strict adherence to optimized protocols and rigorous long-term anticoagulation, especially for multiple prostheses where INR targets are higher.

Differential diagnosis with pannus formation, a fibrotic ingrowth that can also restrict prosthetic leaflet motion, was considered. However, the acute presentation, subtherapeutic INR, elevated D-dimers, and rapid response to thrombolysis strongly supported a thrombotic rather than pannus etiology.

Overall, the convergence of meta-analyses, prospective studies, and contemporary cohorts positions thrombolysis as a safe, effective, and life-saving alternative to urgent surgery in carefully selected patients. In centers with structured low-dose or ultra-slow infusion protocols and robust follow-up, fibrinolysis should be actively considered as part of the therapeutic armamentarium. Future randomized trials are warranted to refine selection criteria and harmonize recommendations.

## Conclusions

Thrombolysis represents a valuable therapeutic option for obstructive PVT in carefully selected patients, particularly when surgery is contraindicated or declined. While redo surgery remains the traditional standard, it carries substantial perioperative risk. Evidence from meta-analyses and prospective series demonstrates that optimized thrombolytic protocols can achieve high success rates with acceptable safety.

This case reinforces those observations by illustrating successful recovery after tenecteplase administration under multidisciplinary monitoring. However, as a single case, it should be viewed as hypothesis-generating rather than conclusive. The decision between surgery and thrombolysis must always be individualized, integrating hemodynamic status, valve position, and patient-specific risk-benefit assessment.

Overall, this report supports the growing body of evidence favoring thrombolysis as a safe and feasible option in selected patients, provided treatment follows standardized regimens and rigorous anticoagulation management thereafter.

## References

[1] Jones JM, O'kane H, Gladstone DJ (2001). Repeat heart valve surgery: risk factors for operative mortality. J Thorac Cardiovasc Surg.

[2] Karthikeyan G, Senguttuvan NB, Joseph J, Devasenapathy N, Bahl VK, Airan B (2013). Urgent surgery compared with fibrinolytic therapy for the treatment of left-sided prosthetic heart valve thrombosis: a systematic review and meta-analysis of observational studies. Eur Heart J.

[3] Bonou M, Lampropoulos K, Barbetseas J (2012). Prosthetic heart valve obstruction: thrombolysis or surgical treatment?. Eur Heart J Acute Cardiovasc Care.

[4] Chopard R, Vidoni C, Besutti M (2024). Surgery versus thrombolytic therapy for the management of left-sided prosthetic valve thrombosis without hemodynamic compromise: a systematic review and meta-analysis. J Am Heart Assoc.

[5] Otto CM, Nishimura RA, Bonow RO (2021). 2020 ACC/AHA guideline for the management of patients with valvular heart disease: a report of the American College of Cardiology/American Heart Association Joint Committee on clinical practice guidelines. J Am Coll Cardiol.

[6] Vahanian A, Beyersdorf F, Praz F (2022). 2021 ESC/EACTS guidelines for the management of valvular heart disease. Eur Heart J.

[7] Roudaut R, Serri K, Lafitte S (2007). Thrombosis of prosthetic heart valves: diagnosis and therapeutic considerations. Heart.

[8] Guner A, Kalcik M, Gursoy MO, Gunduz S, Ozkan M (2018). How to perform and manage low-dose and slow/ultra-slow tissue type plasminogen activator infusion regimens in patients with prosthetic valve thrombosis. J Thromb Thrombolysis.

[9] Özkan M, Gündüz S, Gürsoy OM (2015). Ultraslow thrombolytic therapy: a novel strategy in the management of PROsthetic MEchanical valve Thrombosis and the prEdictors of outcomE: The Ultra-slow PROMETEE trial. Am Heart J.

[10] Sadeghipour P, Salehi M, Kohansal E (2024). Ultraslow low-dose thrombolytic therapy in patients with acute mechanical prosthetic heart valve thrombosis - a prospective cohort study. Int J Cardiol.

[11] Soria Jiménez CE, Papolos AI, Kenigsberg BB (2023). Management of mechanical prosthetic heart valve thrombosis: JACC review topic of the week. J Am Coll Cardiol.

[12] Sharif Khan H, Ijaz Z, Ali M (2020). Clinical outcomes of mechanical prosthetic valve thrombosis. Cureus.

[13] Özkan M, Çakal B, Karakoyun S (2013). Thrombolytic therapy for the treatment of prosthetic heart valve thrombosis in pregnancy with low-dose, slow infusion of tissue-type plasminogen activator. Circulation.

